# Autophagy Regulated by Gain of Function Mutant p53 Enhances Proteasomal Inhibitor-Mediated Cell Death through Induction of ROS and ERK in Lung Cancer Cells

**DOI:** 10.1155/2019/6164807

**Published:** 2019-01-06

**Authors:** Heena Saini, Ifrah Hakeem, Sudeshna Mukherjee, Shibasish Chowdhury, Rajdeep Chowdhury

**Affiliations:** Department of Biological Sciences, Pilani Campus, BITS, Pilani, Rajasthan 333031, India

## Abstract

Mutations in p53, especially gain of function (GOF) mutations, are highly frequent in lung cancers and are known to facilitate tumor aggressiveness. Yet, the links between mutant GOF-p53 and lung cancers are not well established. In the present study, we set to examine how we can better sensitize resistant GOF-p53 lung cancer cells through modulation of cellular protein degradation machineries, proteasome and autophagy. H1299 p53 null lung cancer cells were stably transfected with R273H mutant GOF-p53 or wild-type (wt) p53 or empty vectors. The presence of R273H-P53 conferred the cancer cells with drug resistance not only against the widely used chemotherapeutic agents like cisplatin (CDDP) or 5-flurouracil (5-FU) but also against potent alternative modes of therapy like proteasomal inhibition. Therefore, there is an urgent need for new strategies that can overcome GOF-p53 induced drug resistance and prolong patient survival following failure of standard therapies. We observed that the proteasomal inhibitor, peptide aldehyde N-acetyl-leu-leu-norleucinal (commonly termed as ALLN), caused an activation of cellular homeostatic machinery, autophagy in R273H-P53 cells. Interestingly, inhibition of autophagy by chloroquine (CQ) alone or in combination with ALLN failed to induce enhanced cell death in the R273H-P53 cells; however, in contrast, an activation of autophagy by serum starvation or rapamycin increased sensitivity of cells to ALLN-induced cytotoxicity. An activated autophagy was associated with increased ROS and ERK signaling and an inhibition of either ROS or ERK signaling resulted in reduced cytotoxicity. Furthermore, inhibition of GOF-p53 was found to enhance autophagy resulting in increased cell death. Our findings provide novel insights pertaining to mechanisms by which a GOF-p53 harboring lung cancer cell is better sensitized, which can lead to the development of advanced therapy against resistant lung cancer cells.

## 1. Introduction

Non-small cell lung carcinoma (NSCLC) is a collective term for a group of lung cancers which affects both smokers and nonsmokers. It represents approximately 85% of all lung cancers. In India, more than one million new cases arise every year with a burgeoning incidence of NSCLC reported annually. In more than 50% of NSCLC patients, p53 is arguably the most frequent target for genetic alterations associated with poor prognosis and relatively more chemoresistance [1]. Accumulating evidences show that a vast majority of p53 mutations are missense that results in production of a stable, full-length mutated protein carrying only single amino acid substitution. These mutations not only annul p53's tumor-suppressive function but also in certain instances can endow mutant proteins with neomorphic properties described as mutant GOF-p53 which can contribute actively to various stages of tumor progression and to increased resistance to chemotherapy. In this regard, the central DNA-binding domain of p53 spans the most conserved region composed of a vast number of these missense mutations and among these the hot spot residues occur with unusually high frequency [2–4]. p53 missense mutations in the hot spot region can generally be classified as DNA contact (or class I) mutants, like R273H-p53, which normally make direct contact with target DNA sequences and conformational (or class II) mutants, like R175H-p53, which disrupt the structure of the p53 protein partially or completely, thus altering its function [5, 6]. R175H-P53 and R273H-P53, being the most frequently occurring GOF mutations in cancer cells, were observed to induce resistance to chemotherapeutic agents in multiple cancer cell types [7, 8]. Interestingly, few reports suggest that, unlike wild-type (wt) p53, mutant p53 can escape MDM-2-dependent proteasomal degradation and hence accumulate stimulating the oncogenic effect [9]. Thus, how to effectively promote degradation of GOF-p53 and sensitize cancer cells, thus reducing drug resistance, is an important question to be investigated.

Autophagy is a well-established self-degradation process that degrades and recycles numerous intracellular cytoplasmic constituents to maintain homeostasis. However, in a cancerous state, autophagy is known to play a paradoxical role by either activating cell death and inhibiting tumor progression or promoting cell survival and later stages of cancer progression [10]. Substantial evidences show that different forms of autophagy, for example, macroautophagy and chaperone-mediated autophagy, are implicated in the depletion of stable, mutant p53 isoforms [11]. Functional involvement of mutant p53 in the regulation of autophagy and in turn being regulated by the cellular degradation system in cancer cells and identification of associated molecular mechanisms governing it are still incompletely understood. Deciphering the details of these interactions can provide clues to appropriate sensitization of resistant cancer cells.

A growing number of studies suggest that both intracellular degradation pathways, for example, ubiquitin proteasome pathway (UPS), and autophagy are mechanistically and functionally linked such that blockage to either one can lead to upregulation of the other in a way that remains yet to be clarified [12]. For example, proteasomal inhibition can enhance the load of misfolded proteins and can trigger autophagy as a compensatory mechanism for their degradation [13]. However, autophagy, serving as an essential mechanism to cope with cellular stresses, may directly contribute to survival of cancer cells exposed to proteasomal inhibitors and, hence, in consequence, might reduce effectiveness of therapy. Therefore, an inhibition of autophagic flux after induction of prosurvival autophagy has often been utilized as a strategy to sensitize multiple cancer cell types [14]. Based on these findings, clinical trials are currently ongoing investigating autophagy inhibition in conjunction with anticancer therapies, like bortezomib. However, conversely, an overactivation of autophagy can also act as a bona fide death inducer or death effector, upstream of other death pathways, like apoptosis [15]. Based on the above considerations, there might be physiological situations in which autophagy protects death or, in contrary, kills cancer cells that would have otherwise manifested prolonged survival. Because autophagy plays an important roles in cell fate, it is therefore critical to understand the mechanism by which autophagy interacts with and affects cell survival or death machinery, which might allow new avenues to effectively sensitize resistant cancer cell types.

Taking this into consideration, in this study, we exposed GOF-R273H-P53 cells to proteasomal inhibitor ALLN and observed its effect on autophagy. A ROS-dependent autophagy was found to be induced in the R273H-P53 cells, which acted as a prodeath mechanism. Furthermore, we observed that GOF-p53 serves to mitigate cell death induced by autophagy in the lung cancer H1299 cells. Our study provides novel insights into modes of sensitization of resistant NSCLC cells harboring GOF-R273H mutant P53.

## 2. Materials and Methods

### 2.1. Chemicals and Reagents

ALLN (#sc-221236), pifithrin-alpha (PFT-*α*, #sc-45050), and rapamycin (Rapa, #sc-3504A) were purchased from Santa Cruz Biotechnology. CDDP (#232120) was obtained from MERCK. 2′,7′-dichlorofluorescin diacetate (DCFDA, # D6883), monodansylcadaverine (MDC, # D4008), CQ (#C6528), and propidium iodide (PI; #P4864) were purchased from Sigma; N-Acetyl-L-cysteine (NAC, #47866) and 3-(4, 5-dimethylthiazol-2-yl)-2,5-di-phenyltetrazolium bromide (MTT, #33611) were obtained from SRL. Geneticin (G418, # 10131-035), FITC conjugated Annexin V (#A13199), Annexin V binding buffer (#V13246), and LysoTracker Green DND-26 (# L7526) were procured from Thermo Fisher Scientific. Lipofectamine 3000 was from Invitrogen (#L3000-001). The MAPK inhibitor, U0126, and gene specific primary and secondary antibodies were obtained from Cell Signaling Technology (CST, USA). pCMV-Neo-Bam p53 wt (Addgene Plasmid #16434), pCMV-Neo-Bam p53 R273H (Addgene Plasmid #16439), and pCMV-Neo-Bam Empty Vector (Addgene Plasmid # 16440 ) were a gift from Bert Vogelstein.

### 2.2. Cell Culture

The non-small cell lung carcinoma cell line, H1299, was a kind gift from Dr. Sanjeev Das (NII, New Delhi). MCF-7 and MDA-MB-468 cells were procured from NCCS (Pune, India); HCT116 (p53 null) cells were obtained from Wogan Lab (MIT, USA); HT-29 and SW480 cells were a gift from Dr. Susanta Roychoudhury, IICB-Kolkata. Cells were cultured at 37°C, 5% CO_2_ in Dulbecco's modified minimal essential medium supplemented with 10% fetal bovine serum and 1% penicillin-streptomycin mixture [16]. Cells were grown to 70-80% confluency prior to treatments. Trypsin-EDTA solution (0.05%) was used for detachment of cells. Stable transfected cells were maintained in 600 *μ*g/ml of G418.

### 2.3. Preparation of Cell Lines Stably Expressing GOF Mutant and wt-p53

P53 null H1299 cells were cultured in six-well plate and transfected with either 2 *μ*g of pCMV-Neo-Bam p53-wt or pCMV-Neo-Bam p53-R273H or pCMV-Neo-Bam Empty Vector (EV) purified plasmid with Lipofectamine 3000 according to manufacturer's instructions. Around 24 h after transfection, the cells were transferred to a 10 cm dish and selected for transfection positivity by geneticin (600 *μ*g/ml) selection. Transfected cells were maintained for several days under G418 pressure. Nonsurviving cells were washed off with PBS and fresh media with G418 was added. This was done till the time cell colonies were obtained. Random colonies were selected and allowed to grow in a new culture dish under G418 pressure. Cells were grown till confluency and then stable transfection of wild-type or R273H-P53 vector was confirmed by immunoblotting against p53 antibody.

### 2.4. Analysis of Cytotoxicity

*In vitro* cytotoxicity was performed using 3-(4,5-dimethylthiazol-2-yl)-2,5-di-phenyltetrazolium bromide (MTT) assay, following methods previously described in Chowdhury et al's work [17]. Briefly, H1299 (null P53/EV), H1299/wt (wt P53), and H1299/R273H (R273H P53) cells were cultured in 96-well plates. After overnight culture of cells, they were treated with specific drugs for a stipulated period of time. Following that, MTT was added to each well and incubated for 4 h. Viable cells form formazan crystals with MTT, which were solubilized in DMSO, and readings were obtained at 570 nm with a differential filter of 630 nm using Multiskan GO microplate spectrophotometer. Percentage of viable cells was calculated using the following formula: viability (%) = (mean absorbance value of drug-treated cells)/(mean absorbance value of the control) x 100.

### 2.5. Caspase Assay

For measurement of caspase activity, 2.5 × 10^5^ cells/well were seeded in 6-well plate and exposed to IC_50_ dose of CDDP for 48 h. Caspase-3 colorimetric protease assay kit (Invitrogen) was used to measure caspase-3 activity following procedure described elsewhere [17]. Briefly, protein was extracted using RIPA buffer; concentration was determined by Bradford assay and then equal amount (60 *μ*g) of protein was added to microtiter plates with caspase-3 substrate (Ac-DEVD-pNA). The concentration of the p-nitroaniline (pNA) released from the substrate was calculated from the absorbance values at 405 nm.

### 2.6. Analysis of DNA Fragmentation

Apoptosis was evaluated by fragmented genomic DNA forming DNA ladders (short fragments of ~200 base pairs) on agarose gel [18]. To analyze DNA ladder formation, null P53 and R273H-P53 cells were seeded in 6 cm dishes at a density of 5× 10^5^ cells/plate and treated with CDDP for 48 h. DNA was extracted using Invitrogen apoptotic DNA ladder detection kit and ladder formation was analyzed on 1% agarose gel. A DNA marker was run parallel to the samples.

### 2.7. RNA Isolation and Real-Time PCR (RT-PCR)

Total RNA was isolated using TRIzol reagent. GeneSure First Strand cDNA Synthesis kit was used for complementary DNA (cDNA) synthesis. Templates were amplified using gene-specific primers for ABCB1 taking GAPDH as housekeeping control and detected using SYBR Green Supermix in CFX Connect RT-PCR System (Bio-Rad) [16]. The primers used and their sequences are given as follows: GAPDH, forward 5′-GCA CCG TCA AGG CTG AGA AC-3′ and reverse 5′-TGG TGA AGA CGC CAG TGG A-3′; ABCB1, forward 5′-GGG ATG GTC AGT GTT GAT GGA-3′ and reverse 5′-GCT ATC GTG GTG GCA AAC AAT A-3′. The relative RNA expression was calculated using Pfaffl's method [19].

### 2.8. DAPI Staining of Nucleus

4′-6-Diamidino-2-phenylindole (DAPI) stain was used to detect apoptotic nuclei [18]. Cells grown on coverslips were either untreated or treated with drug for 48 h. Coverslips were then withdrawn and washed with PBS and cells were fixed in formaldehyde for 10 min at room temperature. Coverslips were mounted on a glass slide with antifade DAPI. Nuclear morphology was further observed by fluorescence microscopy (Olympus, Japan).

### 2.9. Annexin V/Propidium Iodide (PI) Staining

Cells were seeded on 6 cm dishes at a density of 5 × 10^5^ cells and thereafter exposed to various treatments. After that the cells were harvested, washed with PBS, and resuspended in 500 *μ*L of 1X binding buffer. Following that, 4 *μ*l of Annexin V-FITC and 10 *μ*l of PI were added. Cells were then incubated in the dark for 20 min and samples were then acquired using flow cytometer (Cytoflex, Beckmann Coulter) [17]. CytExpert software was used to analyze the acquired data. To detect both early and late apoptotic cells, the percentage of cells in lower and upper right (LR and UR) quadrant representative of only Annexin V and both Annexin V-PI positive cells, respectively, were counted. Percentage of apoptotic cells is represented through bar diagram.

### 2.10. Monodansylcadaverine (MDC) Staining of Autophagic Vacuoles

MDC, an autophagolysosomal marker, was used to analyze autophagy induction. Cells were grown over coverslips and the following day drug treatment was made. The cells were then incubated in the dark for 10 min with 0.05 mM MDC at 37°C. Thereafter, the coverslips with cells were washed and mounted with antifade DAPI. MDC punctate dots were analyzed under fluorescence microscope. For fluorimetric measurements, cells were grown in 6-well plate. After treatment, cells were labelled with MDC for 10 min followed by PBS wash and then collected in 10 mM Tris-HCl (pH 8.0) containing 0.1% Triton X-100 [20, 21]. Intracellular MDC was assessed by fluorescence photometry (excitation 380 nm and emission 525 nm) on a microplate reader (Fluoroskan Ascent™). Change in MDC fluorescence with respect to control is expressed as fold change.

### 2.11. LysoTracker Green (LTG) Staining of Acidic Vesicles

LTG constituting a fluorophore linked to a weak base is a fluorescent acidotropic probe, used for labelling and tracking acidic organelles in live cells. Cells were cultured overnight on coverslips and then exposed to different concentration of ALLN for 48 h. After treatment, the media was removed, the cells were washed with PBS, and thereafter LTG was added (0.05 *μ*M). Cells were then incubated for 20 min in CO_2_ incubator. Fluorescence intensity of LTG was observed under a fluorescence microscope and compared to untreated control.

### 2.12. Measurement of Intracellular ROS

Cells were seeded at a density of 9x10^3^ in 96-well plates and exposed to treatments. NAC, a ROS scavenger (5 mM), was added 1 h prior to treatments. After treatment, the cells were washed with PBS and then incubated in 100 *μ*l of working solution (10 *μ*M) of DCFDA at 37°C for 30 min. Fluorescence was measured using a microplate reader (Fluoroskan Ascent) at 485 nm excitation and 530 nm emission [18].

### 2.13. Immunoblotting

Immunoblotting was performed following protocols described elsewhere [22]. Cells were grown in 10 cm dishes. Following treatment for specific time, cells were lysed in RIPA buffer (Sigma-Aldrich), and the protein concentration was estimated using Bradford reagent. Then, 5X gel loading dye was added to the lysates followed by heat denaturation (100°C for 10 min). Proteins were then loaded in denaturing polyacrylamide gels and transferred to polyvinylidene fluoride membranes. Skimmed milk (5%) was used for blocking. The blots were then probed or reprobed with specific primary antibodies and detected using enhanced chemiluminescence detection on ChemiDoc (Bio-Rad) [14]. The primary antibodies used were as follows: Atg-5 (CST; D1G9), Atg-3 (CST; 3415), LC3-II (CST; D11), phospho-MAPK (ERK) (CST; Rabbit mAb #4370), and p53 (SCBT; DO-1). *β*-actin (SCBT; sc69879) was used as loading control. The secondary antibodies used were horseradish peroxide-conjugated goat anti-rabbit and goat anti-mouse IgG at dilution of 1:10,000 and 1:20,000, respectively. The expression levels were densitometrically quantified using ImageJ software and normalized to the control.

### 2.14. Statistical Analysis

The obtained data were analyzed using GraphPad Prism software version 5.0. Effect of treatment in comparison to control was statistically determined using one-way or two-way ANOVA. The Bonferroni method was used to analyze multiple comparisons. Throughout the text, the representative images are of experiments done in multiples. Data are represented in mean ± SEM. The symbols in parenthesis denote the following: *∗* compared to control, # compared to ALLN or R273H, not significant (ns) p > 0.05;^*∗*/#^p ≤ 0.05; ^*∗∗*/##^p ≤ 0.01; ^*∗∗∗*/###^p ≤ 0.001.

## 3. Results and Discussion

### 3.1. R273H-P53 Cells Exhibit Increased Drug Resistance Compared to Null or wt-p53 Cells

**Figure 1:**
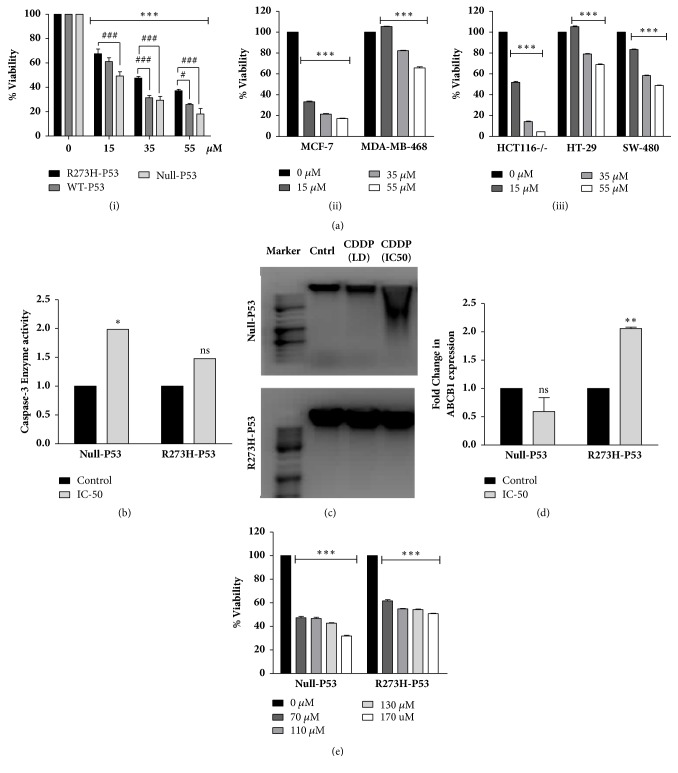
GOF-R273H-p53 mutation imparts drug resistance. (a) Cell lines with varied p53 status were exposed to different doses of cisplatin. Cell viability was analyzed after 48 h of treatment through MTT assay. Untreated samples were taken as control. *∗* and # indicate significant difference compared to untreated cells and R273H-P53 cells, respectively. (b) Fold change in caspase-3 enzyme activity was measured following CDDP treatment at IC-50 dose for 48 h in H1299 (null-P53) and H1299-R273H-P53 stable transfected cells. Level of caspase-3 activity in untreated control was taken as arbitrary unit “1.” (c) DNA fragmentation was measured on agarose gel. Null-P53 and R273H-P53 cells were treated with CDDP at low dose (LD) and IC-50 dose for 48 h; DNA was extracted and run on an agarose gel. A DNA marker was run alongside the samples. (d) Real-time PCR showing expression of ABCB1 mRNA levels upon exposure of null-P53 and R273H-P53 cells to CDDP (IC-50) for 48 h. (e) Null-P53 and R273H-P53 cells were given different doses of 5-FU and cross resistance was analyzed through MTT assay. *∗* indicates significant difference compared to untreated cells.

In NSCLC patients, p53 status shows no prognostic significance in the absence of adjuvant chemotherapy; however, after undergoing treatment with cisplatin, a reduced disease-free interval and overall survival are seen bearing a GOF-p53 protein [23]. Given the importance of GOF-p53 in NSCLCs, in this study, we prepared a stable transfected GOF-R273H-P53 NSCLC cell model aimed at understanding* modus operandi* of resistance and develop an effective strategy for sensitizing GOF p53 cells, which is elusive till date. P53 null H1299 cell line was stable transfected with empty vector (EV), wt-P53, or R273H-P53. Stable transfection was confirmed by immunoblotting against p53 antibody (Supplementary Figure 1.a). The cells were then exposed to varying concentrations of CDDP (a standard chemotherapy for NSCLC patients) for 48 h. Similarly other cancer cell types possessing wt-P53 (MCF7), null-P53 (HCT116), or R273H-P53 (MDA-MB-468, HT-29, and SW480) were also exposed to various doses of CDDP. Interestingly, in all the cell types studied, GOF-p53 cells showed significantly decreased sensitivity to CDDP compared to wt-P53 harboring cells demonstrating resistance (Figures 1(a)(i)–1(a)(iii)). Importantly, R273H-P53 cells showed less sensitivity to CDDP compared to either parental H1299 null P53 cells or EV stable transfected cells (Figure 1(a)(i), Supplementary Figure 1.b, and Supplementary Figure 1.c); the sensitivity of EV cells to cisplatin was comparable to null cells. We hence compared caspase-3 activity upon CDDP treatment between H1299 null P53 (IC_50^~^_ 15 *μ*M) and R273H-P53 (IC_50^~^_ 30 *μ*M) cells through ELISA based method. As expected, R273H-P53 cells showed significantly decreased enzymatic activity compared to null P53 cell type (Figure 1(b)). Similar results were obtained in DNA fragmentation study (Figure 1(c)). Since R273H-P53 cells showed a marked increase in resistance compared to null or wt-P53 cell types, we further confirmed it by analyzing mRNA expression of ABCB1, which showed a substantially increased expression upon CDDP treatment in R273H-P53 stable transfected cells compared to control (Figure 1(d)). Based on the above experimental evidences, it is clear that R273H-P53 cells show resistance to CDDP, compared to null or wt-P53 cells. However, to analyze that the resistance is a generalized phenomenon across multiple drug types or is purely specific to CDDP, we evaluated cross resistance of R273H-P53 cells to other conventionally used anticancer drugs like 5-FU or methotrexate (data not shown). As evident from Figure 1(e), R273H-P53 cells were less sensitive to 5-FU as well. Collectively, these observations suggest that null-P53 cells acquire drug-resistant characteristics upon stable transfection of R273H-P53 vector in NSCLC cells.

### 3.2. P53 Positive Cells Are More Sensitive to Proteasomal Inhibition

**Figure 2:**
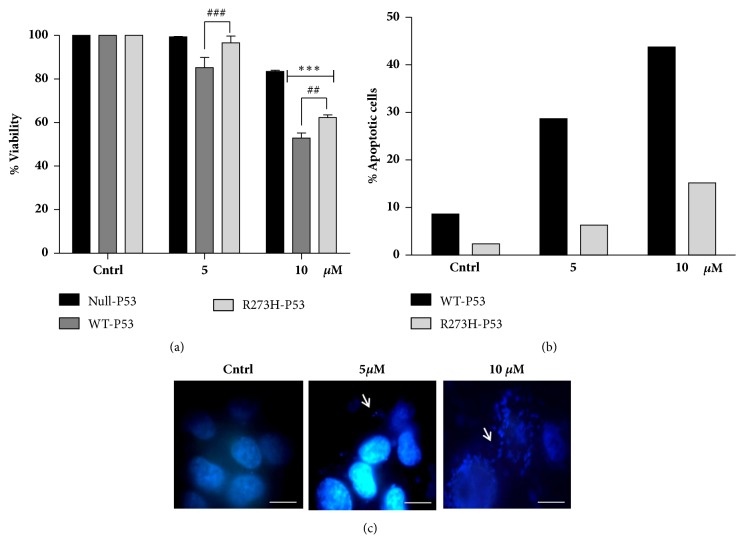
Proteasomal inhibitor (ALLN) induces apoptosis. (a) Null-P53, wt-P53, and R273H-P53 cells were exposed to varying doses of ALLN. After 48 h of exposure, cell viability was measured through MTT assay. Untreated samples were taken as control. *∗* and # indicate significant difference compared to Null-P53 and R273H-P53 cells, respectively. (b) Percentage of apoptotic cells was measured through flow cytometry using Annexin V/PI staining and compared between wt-P53 and R273H-P53 cells upon ALLN treatment. (c) R273H-P53 cells were either untreated or treated with ALLN for 48 h and then stained with DAPI. Nuclear fragmentation or condensation after treatment is marked with white arrows. The scale bar represents 100 *μ*m.

Targeting the proteasomal degradation pathway is increasingly getting recognized as a promising strategy for cancer therapy [24–26]. Wt-p53 protein is primarily degraded by the UPS pathway; however, mutations in p53 might stabilize this protein and inhibit MDM2 interaction, thereby preventing degradation [27–29]. However, there are reports suggesting that several “hot-spot” p53 mutants like R175H, R248W, or R273H remain sensitive to ubiquitin-mediated degradation [30]. We assumed that proteasomal degradation of GOF-p53 might be context-dependent and shows increased bias towards the nature of inhibitor used. We used ALLN, a well-known proteasome inhibitor that is known to induce apoptosis by virtue of accumulated protein response. ALLN was chosen over the widely used proteasomal inhibitor, bortezomib, because the effects of ALLN are relatively less explored and the use of bortezomib has recently been challenged by severe adverse side effects and resistance [31]. Exposure of ALLN (5 and 10 *μ*M) for 48 h showed more cell deaths in wt-P53/R273H-P53 transfected cells than null (Figure 2(a)) or EV transfected cells (Supplementary Figure 2). Similar results were obtained when cells were treated with another proteasomal inhibitor, MG132 (data not shown). Interestingly, R273H-P53 cells in comparison to wt-p53 showed decreased sensitivity to proteasomal inhibition as well (Figures 2(a) and 2(b)) as evident from cell viability assay or apoptosis assay by Annexin V/PI staining. Importantly, unlike response to conventionally used drugs like cisplatin, both wt-P53 and R273H-P53 cells were more sensitive than the parental null or EV cells, suggesting that the presence of p53 protein provides a therapeutic advantage to targeting strategies based on interference of protein degradation (Figure 2(a)). Nuclear staining by DAPI in R273H cells showing fragmented nucleus, as depicted in Figure 2(c), further confirmed cell sensitization on ALLN treatment. Overall, the above results highlight the importance of targeting the protein degradation machinery in p53 positive cells, though an adjuvant therapy might be essential for R273H-P53 cells as they are more resistant than wt-p53 cells to cell death by protein overload.

### 3.3. Proteasomal Inhibitor, ALLN, Induces Autophagy in GOF R273H-P53 Cells

**Figure 3:**
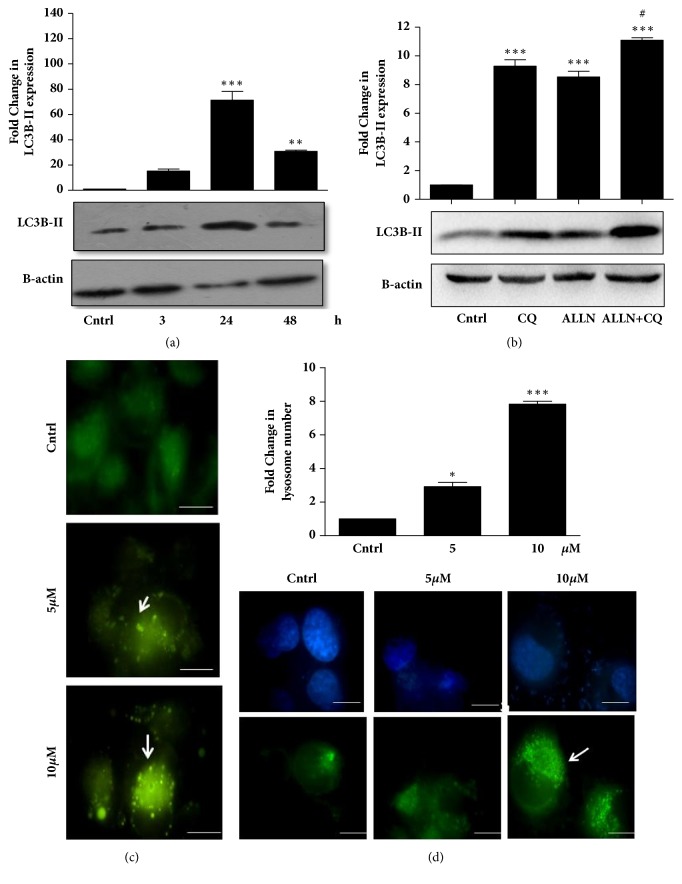
ALLN induces autophagy in GOF R273H-P53 cells. (a) R273H-P53 cells were treated with 10 *μ*M ALLN for different time points and expression of LC3B-II was analyzed through immunoblotting. (b) Immunoblot analysis showing expression of LC3B-II upon 48 h of exposure to CQ, ALLN, and ALLN+CQ in R273H-P53 cells. (c) Fluorescent microscopic images of MDC fluorescence in ALLN-treated (0, 5, and 10 *μ*M; 48 h) R273H-P53 cells. The scale bar represents 100 *μ*m. (d) R273H-P53 cells were treated with ALLN for 48 h and then stained with LTG dye. Green dots representing the lysosomes were counted and represented as bar diagram. The scale bar represents 100 *μ*m.

Two major pathways of degradation maintain protein homeostasis, the UPS, responsible for degrading majority of proteins including many short-lived, denatured, or, in general, damaged proteins, and autophagy, which, by contrast, is mostly responsible for degradation of long-lived proteins [32]. Although UPS and autophagy were initially considered to be largely disconnected pathways, recent advances in understanding of UPS and autophagy have highlighted a strong connection between them. To examine the effect of ALLN on autophagy, R273H-P53 cells were treated with 10 *μ*M ALLN for different time points and LC3B-II (marker for autophagy) protein expression was analyzed. An increase in LC3B-II expression levels indicative of enhanced autophagy was observed with ALLN treatment (Figure 3(a)). However, an increase in LC3B-II protein levels can be resultant of increased autophagy or an inhibition of final step of autophagosome-lysosomal fusion [33]. Hence, to confirm autophagic flux, we checked for the changes in LC3B-II protein levels with or without the lysomotropic agent, CQ. An increased expression of LC3B-II was observed in ALLN+CQ treated samples when compared to only independent treatments indicative of enhanced autophagic flux with ALLN (Figure 3(b)). Autophagy induction was further validated through MDC staining; MDC preferentially accumulates in acidic autophagic vacuoles. MDC fluorescence, represented by the green punctate dots, increased with ALLN treatment in a dose-dependent manner, suggesting an increase in autophagy (Figure 3(c)). Furthermore, an increase in the number of lysosomes is often associated with increased autophagy. We observed an increase in LTG staining with ALLN (Figure 3(d)). The above results are supportive of the fact that proteasomal inhibition by ALLN in R273H-p53 cells activates autophagy. However, in tumor cells, autophagy can act as both a prodeath or prosurvival mechanism in a context-dependent manner [34].

### 3.4. GOF-R273H-p53 Cells Are Sensitive to Autophagy Induction

**Figure 4:**
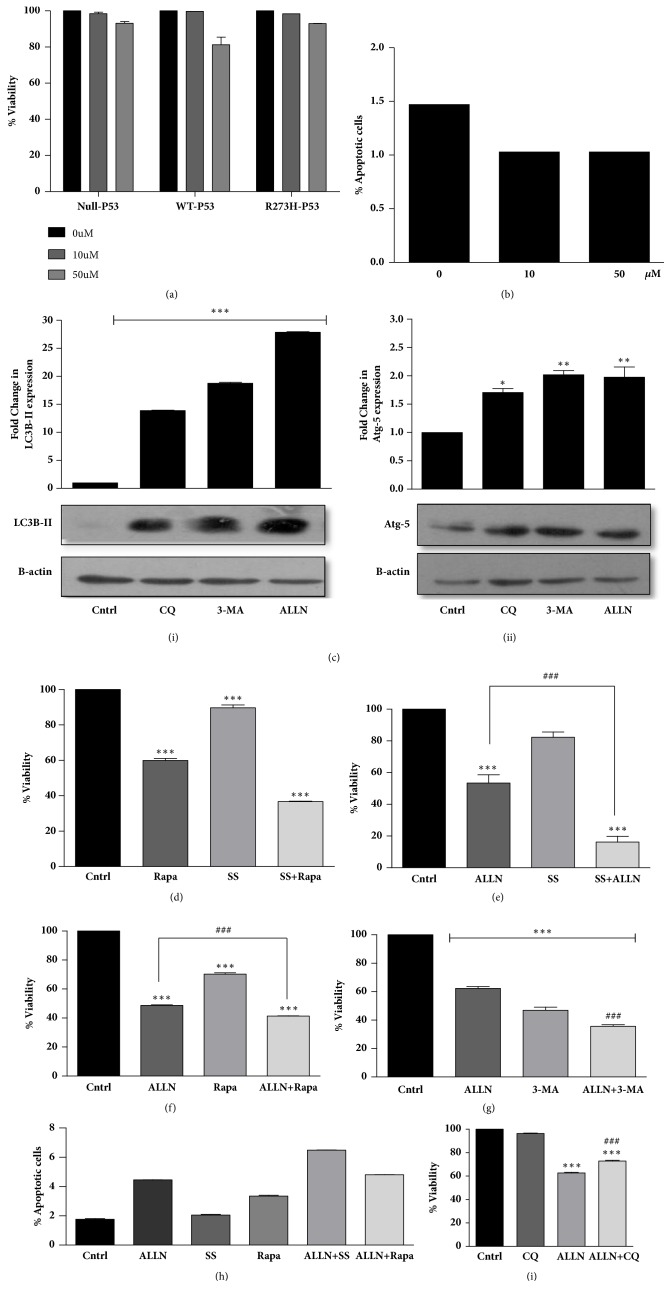
Autophagy induction promotes cell death in R273H-P53 cells. (a) Null-P53, wt-P53, and R273H-P53 cells were exposed to varying doses of CQ. After 48 h of exposure, cell viability was measured through MTT assay. (b) CQ-treated R273H-P53 cells were analyzed for apoptosis induction through Annexin V/PI staining followed by flow cytometry. Fold change is represented through bar diagram. (c) R273H-P53 cells were exposed to CQ (10 *μ*M), 3-MA (5 mM), and ALLN (10 *μ*M) for 48 h and immunoblot analysis was performed for LC3B-II and Atg-5. *∗* indicates significant difference to untreated control. (d) Cell viability in R273H-P53 cells was measured through MTT assay after 48 h of treatments at various combinations, (d) Rapa (500 nM), Rapa + SS, (e) SS, ALLN+SS, (f) Rapa, ALLN+Rapa, (g) 3-MA, 3-MA+ALLN and represented in the form of bar diagram. (h) Apoptosis was analyzed through flow cytometry using Annexin V/PI staining in R273H-P53 cells, after treatment with various autophagy inducers. Percentage of apoptotic cells is represented through bar diagram. (i) Cell viability was measured through MTT assay in R273H-P53 cells after autophagy inhibition in presence or absence of ALLN. *∗* indicates significant difference compared to untreated control, while # indicates significant difference compared to ALLN-treated cells.

Enhanced autophagy has recently been implicated in multiple studies facilitating cancer cell survival under physiological stresses. Hence, we hypothesized that a combination treatment with ALLN (being an autophagy inducer) with CQ (an agent that inhibits lysosomal function) might lead to accumulation of acidic vesicles, thereby blocking autophagic flux, and can act as a potent strategy to sensitize R273H-P53 cells. We initially checked for cytotoxicity inducing property of CQ alone. CQ even at 50 *μ*M was not able to impart any significant cytotoxic effect on R273H-P53 cells as analyzed by MTT assay and also Annexin V/PI staining (Figures 4(a) and 4(b)). Since CQ is a late-stage autophagy inhibitor, we thought of exploring the effect of an early-stage autophagy inhibitor, 3-methyl adenine (3-MA). Interestingly, on the contrary, 3-MA treatment was able to sensitize R273H-P53 cells. As depicted in Supplementary Figure 3, only 3-MA treatment reduced cell viability to less than 50% in R273H-P53 cells. However, as we analyzed the expression of autophagic markers upon 3-MA exposure, we noticed an increased expression of LC3B-II and Atg-5 proteins levels (Figures 4(c)(i) and 4(c)(ii)). We assumed that 3-MA might have autophagy inducing effects as well. Indeed, in corroboration to above, there are previous reports suggesting that prolonged 3-MA treatment can inhibit PI3K-class-I, in turn inhibiting mTOR and activating autophagy [35]. However, results obtained with 3-MA provided us with a hint that, rather than autophagy inhibition, these cells might be more sensitive to autophagy induction. Accordingly, the effect of rapamycin (Rapa), a widely used mTOR inhibitor, and serum starvation (SS), which are known to elicit an autophagic response, was investigated in R273H-P53 cells [36, 37]. Interestingly, R273H-P53 cells treated with autophagy inducers like Rapa or exposed to SS alone or in combination with ALLN had significantly enhanced cytotoxic effects when compared to autophagy inhibition with CQ alongside ALLN (Figures 4(d)–4(h)). In fact, autophagy inhibition with CQ was found to moderately decrease the sensitivity of cells to ALLN (Figure 4(i) and Supplementary Figure 4). Hence, in this study, we suggest that although physiologically relevant levels of autophagy are required for cellular homeostasis maintenance, enhanced autophagy can in turn induce autophagy-dependent cell death. Excessive autophagy has been previously observed in association with various forms of cell death and the term “autophagic cell death” was originally introduced to describe cell death associated with autophagy. However, evidences associating autophagy to cell death in these reports were more circumstantial, and the nature of such death occurring in cancer cells remains poorly defined. We were hence interested in analyzing the modus operandi of cell death observed upon autophagy enhancement.

### 3.5. ALLN-Induced Autophagy Facilitates Cell Sensitization by Regulating ROS Levels

**Figure 5:**
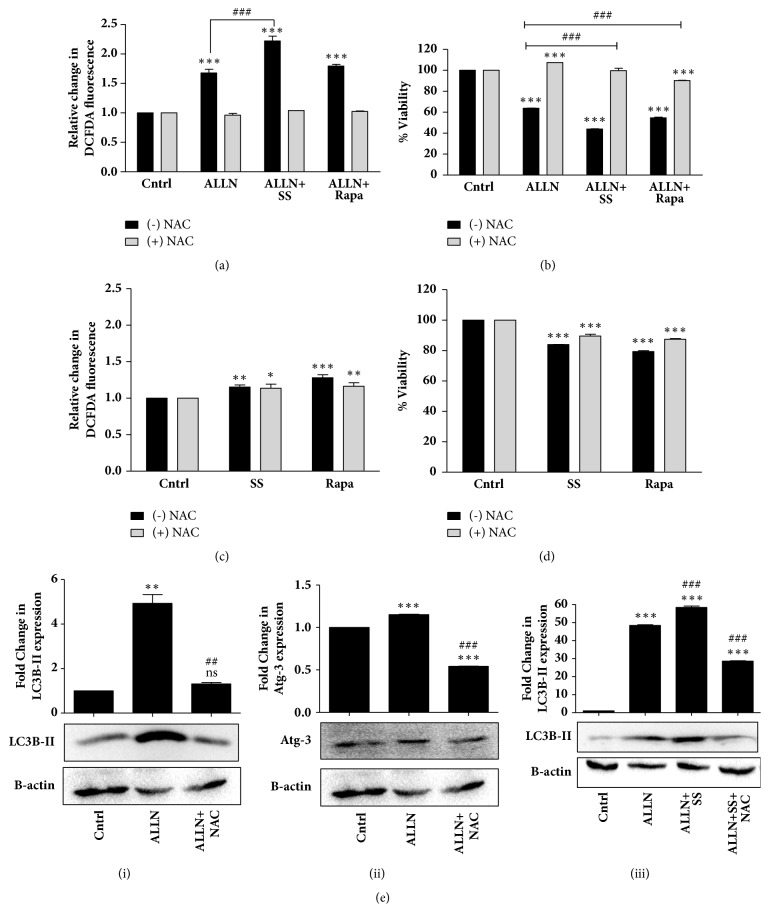
ALLN-induced autophagy sensitizes R273H-P53 cells by increasing ROS levels. (a) R273H-P53 cells were exposed to ALLN, ALLN+SS, and ALLN+Rapa for 48 h. NAC (5 mM) was applied 1 h prior to treatment wherever mentioned. Fold change in ROS levels is represented through bars; untreated control was taken as arbitrary unit “1.” (b) MTT assay was performed to analyze cell viability following exposure of R273H-P53 cells to ALLN and other autophagy inducers; data is represented through bar diagram. (c) R273H-P53 cells were exposed to serum starved media or Rapa (500 nM) for 48 h. Fold change in ROS levels is represented. (d) MTT assay was performed to check cell viability following exposure of R273H-P53 cells to autophagy inducers with/without NAC. *∗* indicates significant difference compared to untreated control, while # indicates significant difference compared to ALLN-treated cells. (e)(i)/(ii)/(iii) R273H-P53 cells were treated with ALLN or ALLN plus SS for 48 h. NAC was given 1 h prior to treatment wherever mentioned. Immunoblotting was thereafter performed for LC3B-II and Atg-3. *∗* indicates significant difference compared to untreated control, while # indicates significant difference compared to ALLN-treated cells.

Generation of ROS through oxidative stress is known to cause cell death; however, the role of oxidative stress in autophagy-induced cell death is relatively unexplored. Conventionally, autophagy serves as a buffer system to control the level of ROS in cells and reduce their toxic effects [38]. However, the connection of ROS with autophagy in our experimental context was found to be different. Intracellular ROS levels measured by DCFDA dye followed by fluorimetric analysis showed a prominent increase with ALLN treatment in R273H-P53 cells (Figure 5(a)). A profound increase in ROS levels was observed upon ALLN treatment in SS condition (Figure 5(a)). Interestingly, pretreatment of cells with the ROS scavenger-NAC resulted in substantial increase in cell viability with only ALLN, ALLN plus SS, or ALLN plus Rapa treated cells, suggesting a direct positive correlation of increase in ROS with cell death (Figure 5(b)). Figures 5(c) and 5(d) represent the status of ROS level and cell viability on Rapa or SS exposure. The experiment was performed with or without NAC pretreatment. The above experiments also confirm that ALLN-induced cytotoxicity is not only autophagy-dependent but is also regulated by an increase in ROS levels. Interestingly, R273H-P53 cells when pretreated with NAC resulted in a decrease in autophagic marker expression, Atg-3 or LC3B-II, suggesting that, in this context, ROS is upstream to autophagy and a quenching of ROS reduces autophagy-induced cell death as well (Figures 5(e)(i), 5(e)(ii), and 5(e)(iii)).

### 3.6. Sensitization of GOF-p53 Is Mediated by ERK along with ROS Accumulation

**Figure 6:**
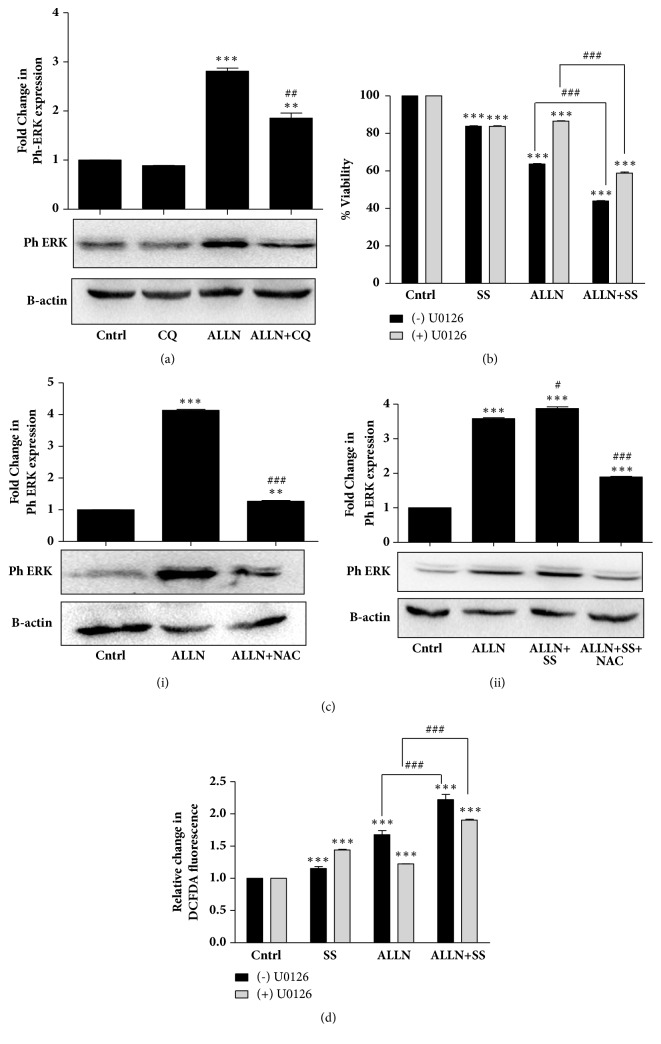
Sensitization of R273H-P53 cells is mediated by ERK signaling and ROS. (a) Immunoblot analysis representing ph-ERK expression upon ALLN and/or CQ treatment in R273H-P53 cells. (b) MTT assay was performed to analyze ALLN and/or serum starvation induced cell death in presence or absence of ERK inhibitor, U0126, given 2 h prior to treatment. Percentage viability is represented in the form of a bar diagram. (c) Immunoblot analysis was performed for ph-ERK expression upon ALLN and/or serum starvation in R273H-P53 cells. NAC was given 1 h prior to treatment wherever mentioned. (d) DCFDA fluorimetric assay measuring intracellular levels of ROS after treatment of R273H-P53 cells with or without U0126, given 2 h prior to ALLN and/or serum starvation for 48 h. *∗* indicates significant difference compared to untreated control, while # indicates significant difference compared to ALLN-treated cells.

Previous reports show that mitogen activated protein kinase (MAPK) signaling pathways are able to modulate autophagy and determine cell fate [39]. There are also a growing number of reports stating that, in certain conditions, the MAPK-ERK can promote cell death [40, 41]. Interestingly, an increase in phospho-ERK indicative of its activation was observed in cells treated with ALLN, which went down with autophagy inhibition by CQ (Figure 6(a)). To confirm the role of ERK in cell death, U0126, a widely used selective inhibitor of the upstream MAP kinase pathway, was used and cell viability was measured in presence of ALLN or ALLN plus SS. Importantly, inhibition of the ERK pathway by U0126 significantly reduced cell death induced by ALLN alone or ALLN plus autophagy induction. This implicates that ERK signaling acts as a prodeath mechanism (Figure 6(b)). ROS, such as hydrogen peroxide, are reported to regulate ERK phosphorylation as ERK-specific phosphatases are sensitive to ROS. Hence, ROS-mediated prolonged ERK activation might be a crucial mechanism regulating cell death [42]. Interestingly, a significant decrease in phospho-ERK levels was observed when ALLN-treated cells were predisposed to NAC (Figures 6(c)(i) and 6(c)(ii)). We also checked for the reverse effect, that is, ROS production upon ERK inhibition. R273H-P53 cells were pretreated with U0126 for 2 h prior to addition of ALLN or ALLN plus autophagy inducers. Interestingly, a drastic decline in level of ROS was observed with ERK inhibition (Figure 6(d)). Collectively, the above findings highlight that R273H-P53 cell sensitization is regulated by ROS-autophagy-ERK signaling loop upon ALLN or ALLN plus autophagy induction.

### 3.7. Induction of Prodeath Autophagy in R273H-P53 Cells Is a p53-Dependent Process

**Figure 7:**
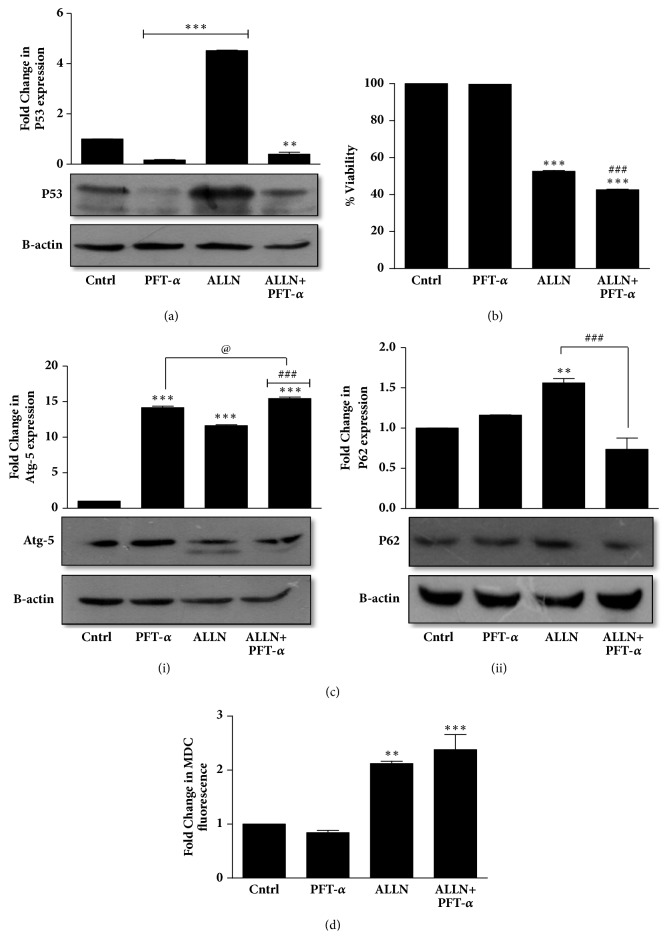
P53 regulates induction of autophagy in R273H-P53 cells. (a) Immunoblot analysis depicting P53 expression upon ALLN treatment with or without 30 min of prior PFT-*α* exposure. (b) MTT assay was performed after ALLN and/or PFT-*α* treatment for 48 h and percentage viability represented in the form of a bar diagram. (c) R273H-P53 cells were exposed to ALLN and/or PFT-*α* for 48 h and autophagy was checked through immunoblot analysis of Atg-5 (i) and P62 (ii). (d) MDC fluorescence assay was conducted after R273H-P53 cells were exposed to ALLN or PFT-*α* or in combination for 48 h. *∗* indicates significant difference compared to untreated control, while # indicates significant difference compared to ALLN-treated cells. “@” indicates significant difference compared to PFT-*α*-treated cells.

**Figure 8:**
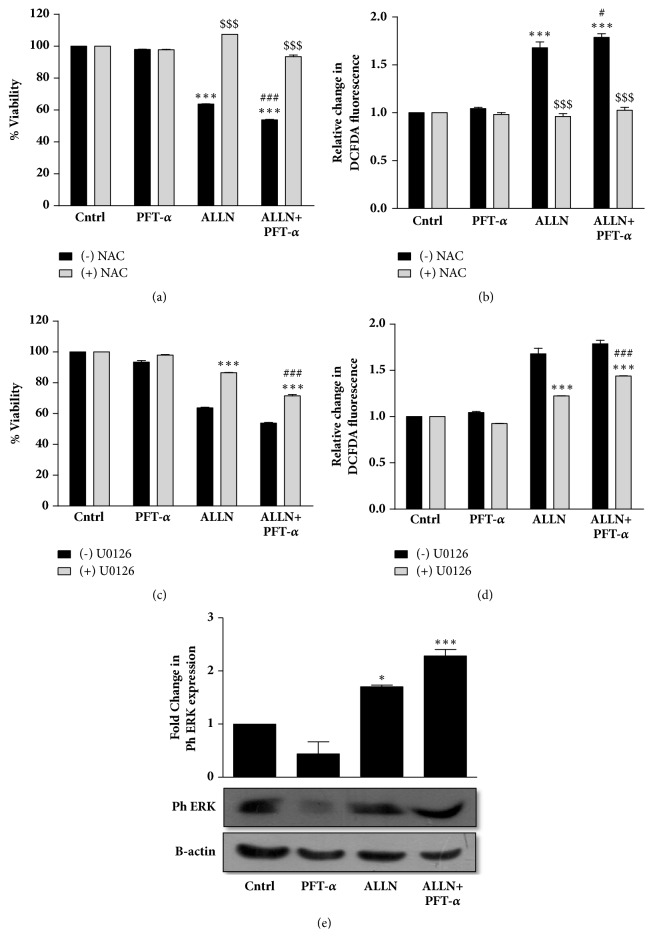
R273H-P53 cells show ROS and ERK accumulation after PFT-*α* treatment. ((a) and (b)) R273H-P53 cells were exposed to ALLN and/or PFT-*α* (30 min prior to ALLN treatment) with and without NAC (1h prior to ALLN treatment) for 48 h and then cell viability and ROS levels were measured through (a) MTT assay and (b) DCFDA fluorimetric assay. ((c) and (d)) Cell viability and ROS levels were analyzed with/without 2 h prior treatment of U0126 and the result is shown in the form of a bar diagram. (e) Immunoblot analysis showing ph-ERK expression on ALLN and/or PFT-*α* treatment in R273H-P53 cells. *∗* indicates significant difference compared to untreated control and # indicates significant difference compared to ALLN-treated cells, while $$$ indicates the statistical difference compared to minus(-) NAC.

Although there are many links that connect autophagy with p53, molecular crosstalk between them is still incompletely understood [43]. One of the earliest studies describing the relationship between mutant p53 and autophagy states that the subcellular localization of mutp53 is the major determinant of autophagy and it is conventionally accepted that p53 inhibits autophagy [44]. On the other hand, in many cells, it is observed that prolonged inhibition of the proteasome leads to its autophagy-mediated degradation of p53, suggesting that autophagy in turn can regulate the stability of p53 protein [11]. In this study, we inhibited p53 with a well-known p53 inhibitor, pifithrin-*α* (PFT-*α*) (Figure 7(a)). Interestingly, a significantly decreased cell viability was obtained with PFT-*α* plus ALLN treatment compared to only ALLN (Figure 7(b)). This suggests that GOF-R273H-p53 inhibition had a positive impact on cell sensitization. We were hence curious to analyze the effects of PFT-*α* on autophagy levels as well. Interestingly, pretreatment of PFT-*α* followed by ALLN exposure caused an increase in Atg-5 levels and a decrease in sequestrosome p62 levels, indicating an enhancement of autophagy (Figures 7(c)(i) and 7(c)(ii)). Enhanced autophagy induction on ALLN plus PFT-*α* treatment was further confirmed by MDC staining which showed an additive effect as well (Figure 7(d)). Furthermore, an increased ROS accumulation was observed with ALLN plus PFT-*α* treatment (Figures 8(a) and 8(b)). ROS scavenger, NAC, successfully reversed the effect substantiating a ROS-dependent phenomenon. Further, as evident from Figure 8(c), U0126 pretreatment followed by ALLN plus PFT-*α* exposure increased the cell viability and decreased accumulated ROS (Figure 8(d)). A significantly increased phospho-ERK level was also observed with ALLN plus PFT-*α* treatment (Figure 8(e)). Taken together, we postulate that GOF-p53 inhibition facilitates cell sensitization by upregulating autophagy and by enhancing ROS and ERK activation.

## 4. Conclusion

For decades, majority of previous studies have focused on understanding protein synthesis, particularly their transcriptional and translational control. While the aspects of protein degradation have been largely overlooked, it is a natural way by which cells clean up proteins that are redundant or have been misfolded or damaged [45, 46]. Inhibition of this protein degradation machinery has been useful previously for treatment of autoimmune diseases and cancer [26, 47]. Especially in particular types of cancer, where protein production is much higher than normal, primarily to meet their overproliferative or secretary demand, an inhibition of proteasomes can cause proteins to pile up, eventually killing the cancer cell [48, 49]. There are already proteasome inhibitors like bortezomib in the clinics for treating Kahler's disease (multiple myeloma). However, the proteasomal inhibitors not only affect the proteasome but also can significantly alter the functioning of another cellular homeostatic machinery like autophagy. The latter is primarily devoted towards maintaining cellular balance of organelles, proteins, and other macromolecules. For example, cancer cells that produce excess unfolded proteins generate high endoplasmic reticulum stress leading to protein removal* via* either the proteasome or autophagy, suggesting that both pathways may be specifically exploited therapeutically. This is further relevant because proteasome inhibition, as in our study, with ALLN leads to compensatory upregulation of autophagy for clearance of proteins, which may provide survival advantage to cells. Given the intense interest in targeting proteasomal degradation and the promise they hold for a multitude of cancer types, it is therefore important to precisely understand the consequences of autophagy induction after inhibition of proteasomal degradation as a cancer therapeutic strategy. In this study, we have demonstrated that cells transfected with GOF mutant p53 show resistance to not only conventional drugs like CDDP or 5-FU but also to ALLN when compared to wt-P53. There was induction of autophagy upon ALLN treatment as well. Since ALLN induces autophagy and the latter may be a tumor survival response, we initially hypothesized that blocking autophagy with CQ, along with ALLN, may initiate cell death or apoptosis. However, despite the proven benefits of lysomotropic agents in cancer clinics in conjunction to autophagy inducers or drugs, in our study, CQ rather than enhancing ALLN-induced cytotoxicity reduced its sensitivity in GOF-R273H-P53 cells. Hence, from these studies, we concluded that, in this context, autophagy does not serve as a mechanism that prolongs cell survival.

**Figure 9:**
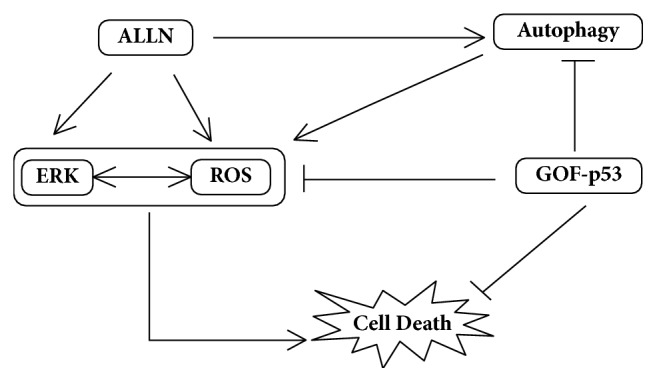
Schematic representation of the probable mechanism involved in proteasomal inhibitor, ALLN induced cell death in R273H-P53 cells.

It is increasingly getting recognized that the well-conserved autophagic machinery may be essential for cell death, at least in certain settings. However, it is controversially discussed in literature whether cells truly die “by autophagy” or in dying cells autophagy is just a bystander or programmed mechanism facilitating apoptosis [50]. Few studies in the past have indeed provided evidence that cells can possess a novel death mechanism that may depend on autophagy [51–53]. However, the nature of stimulus leading to autophagy-dependent cell death has remained poorly defined till date. In this study, we showed that the R273H harboring P53 cells can be better sensitized to proteasomal inhibition by ALLN, by enhancement of autophagy rather than its inhibition. Though autophagy is predominantly thought to play an important protective role in sustaining homeostasis of cancer cells supporting their proliferation, we provide evidences of autophagy as a death promoter in the resistant lung cancer cells. This death was characterized by an enhanced ROS and ERK signaling. We further prove that inhibition of GOF mutant p53 can enhance cell death in the lung cancer cells.  Figure 9 schematically represents the summary of our findings. Currently, there are very few literatures available which identify molecular mechanisms where autophagy is a death enhancer. This signifies the importance of our study. However, more broadly, it still remains to be investigated whether the cell death observed was autosis (that represents a subtype of autophagic cell death) or whether a* bona fide* cell death by autophagy also requires the core apoptotic machinery. Together, our results reveal novel mechanism through which mutant p53 harboring lung cancer cells can be sensitized by exploiting the crosstalk between the cellular homeostatic protein degradation machineries.

## Data Availability

The data used to support the findings of this study are available from the corresponding author upon request.

## Abbreviations

NSCLC: Non-small cell lung carcinoma
UPS: Ubiquitin proteasomal system
ALLN: N-acetyl-leu-leu-norleucinal
PFT-*α*: Pifithrin-alpha
CQ: Chloroquine
ROS: Reactive oxygen species
MDC: Monodansylcadaverine
LC3B-II: Microtubule-associated protein light chain 3-II
SS: Serum starvation
Rapa: Rapamycin.

## Supplementary Materials

Supplementary Materials Supplementary  Fig.  1.a. Stable transfection of WT, R273H-P53, and EV plasmids was confirmed by immunoblotting against P53.** Fig. 1.b. **R273H-P53 and EV transfected cells were exposed to different doses of CDDP followed by MTT assay to analyze the viability. # indicates significant difference compared to R273H-P53.** Fig. 1.c. **Annexin V/PI staining was performed to compare the percentage of apoptotic cells between R273H-P53 and EV transfected cells upon CDDP treatment.** Supplementary Fig. 2. **MTT assay was performed to check for viability between R273H-P53 and EV transfected cells upon ALLN treatment. # indicates significant difference compared to R273H-P53.** Supplementary Fig. 3.** WT-P53 and R273H-P53 cells were exposed to different doses of 3-MA for 48 h and cell viability was analyzed through MTT assay. *∗* indicates significant difference compared to R273H-P53.** Supplementary Fig. 4.** Annexin V/PI staining was performed to compare percentage of apoptotic cells upon exposure to varying doses of ALLN and/or CQ in R273H-P53 cells.
