# Supplementary material for: Autophagy Regulated by Gain of Function Mutant p53 Enhances Proteasomal Inhibitor-Mediated Cell Death through Induction of ROS and ERK in Lung Cancer Cells
Source: J Oncol. 2019 Jan 6;2019:6164807. doi: 10.1155/2019/6164807 (PMC6339715; doi:10.1155/2019/6164807)
Supplement: Supplementary Materials — Supplementary Fig. 1.a. Stable transfection of WT, R273H-P53, and EV plasmids was confirmed by immunoblotting against P53. Fig. 1.b. R273H-P53 and EV transfected cells were exposed to different doses of CDDP followed by MTT assay to analyze the viability. # indicates significant difference compared to R273H-P53. Fig. 1.c. Annexin V/PI staining was performed to compare the percentage of apoptotic cells between R273H-P53 and EV transfected cells upon CDDP treatment. Supplementary Fig. 2. MTT assay was performed to check for viability between R273H-P53 and EV transfected cells upon ALLN treatment. # indicates significant difference compared to R273H-P53. Supplementary Fig. 3. WT-P53 and R273H-P53 cells were exposed to different doses of 3-MA for 48 h and cell viability was analyzed through MTT assay. ∗ indicates significant difference compared to R273H-P53. Supplementary Fig. 4. Annexin V/PI staining was performed to compare percentage of apoptotic cells upon exposure to varying doses of ALLN and/or CQ in R273H-P53 cells. [file 6164807.f1.docx]

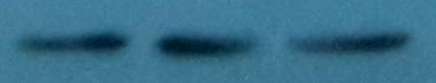

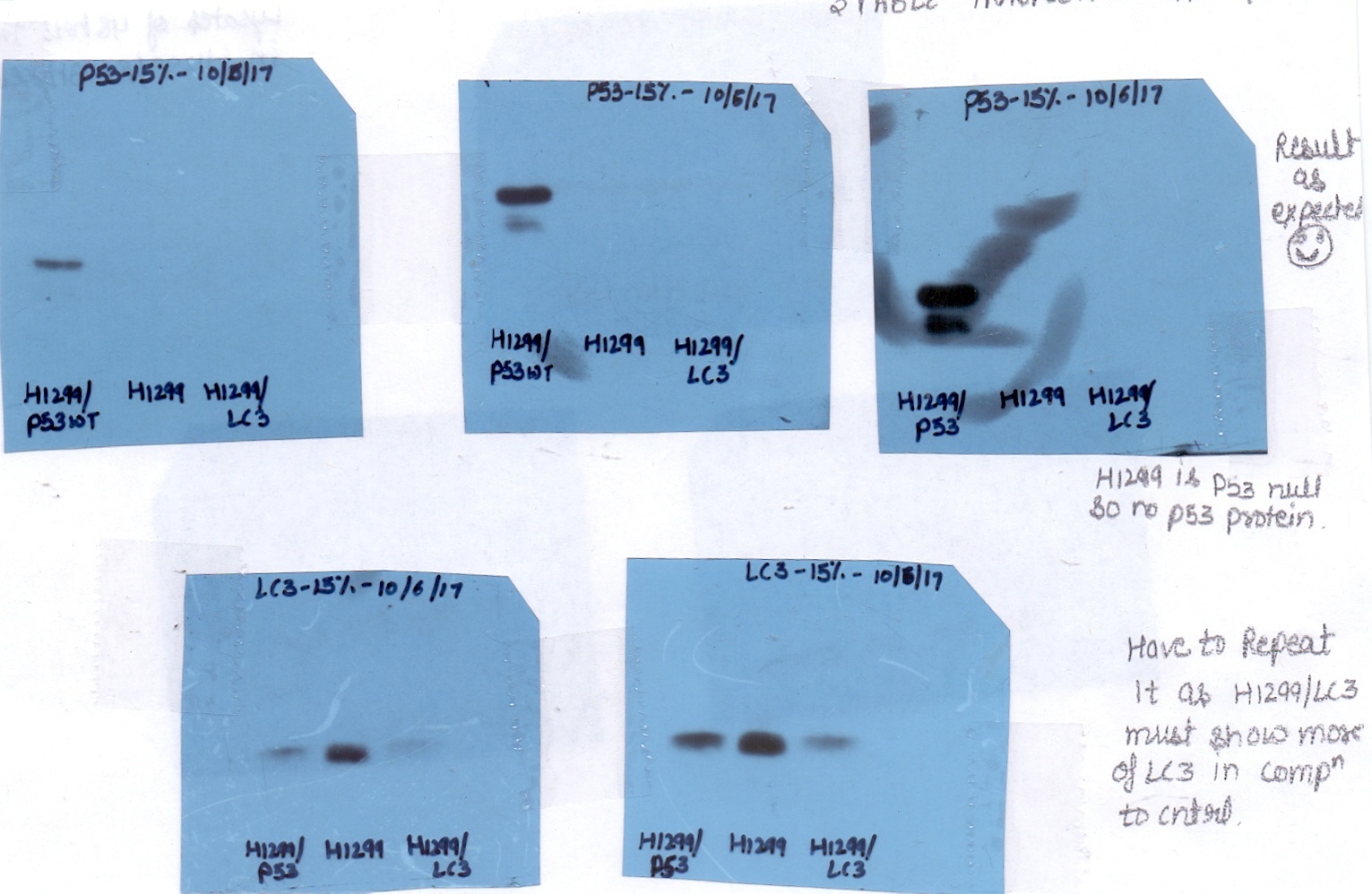

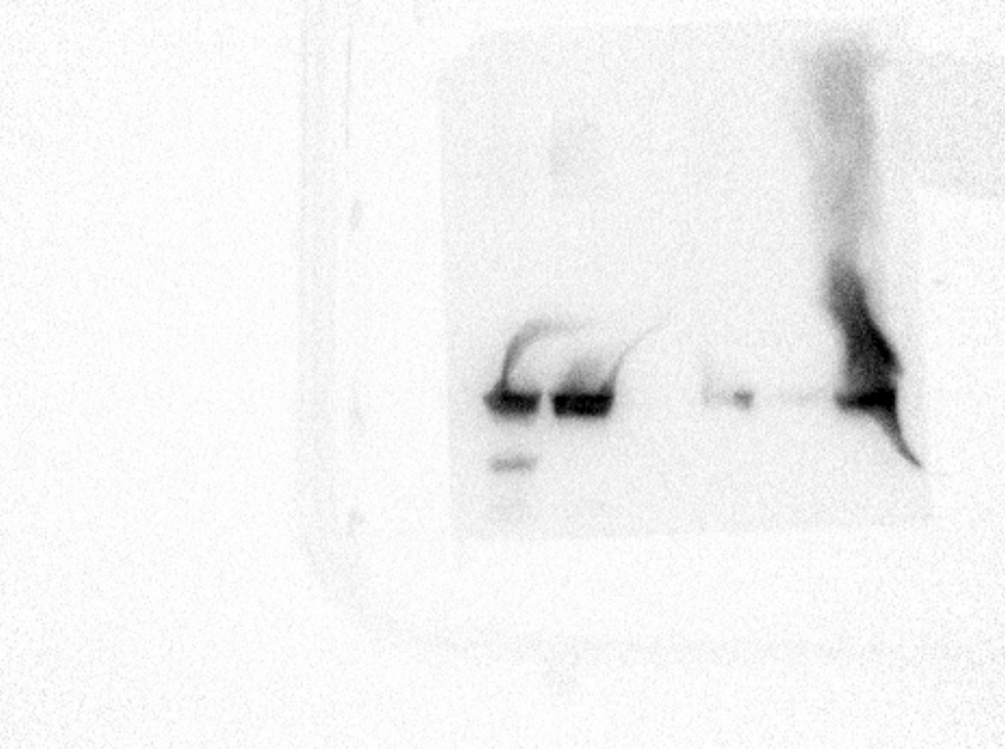

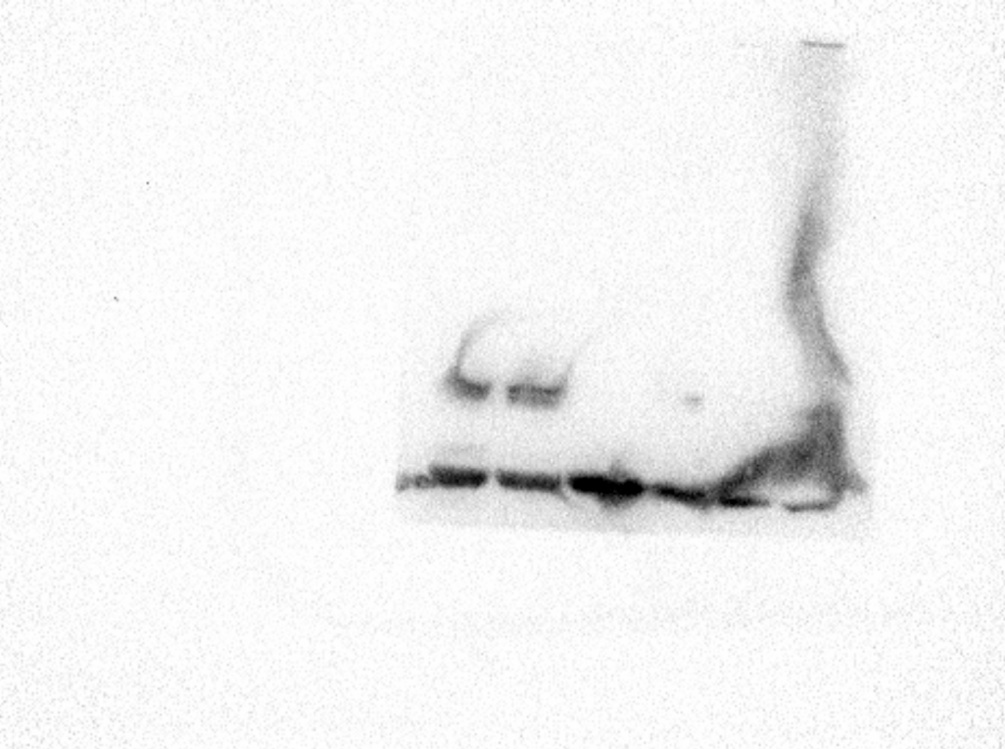


**% Apoptotic cells**

**% Viability**

**% Viability**

**Ctrl ALLN ALLN+SS**

**Ctrl 15 55 µM**

**Ctrl 15 55 µM**

**% Viability**

**P53**

**B-actin**

**H1299/R273H H1299**

**P53**

**B-actin**

**Fig.4.**

**Fig.3.**

**Fig.2.**

**Fig.1c.**

**Fig.1b.**

**Fig.1a.**

**Supplementary Figure:**

**0 5 10 mM**

**% Apoptotic cells**

**H1299/WT H1299/R273H H1299/EV**
